# Bone age is the best predictor of growth response to recombinant human growth hormone in Turner’s syndrome

**DOI:** 10.4103/0971-6866.73400

**Published:** 2010

**Authors:** Nagwa Abdallah Ismail, Nermeen Salah Eldin Metwaly, Fatma Ahmed El-Moguy, Mona Hassan Hafez, Soha M. Abd El Dayem, Tarek Mohamed Farid

**Affiliations:** Department of Pediatrics, National Research Centre, Cairo, Egypt; 1 Department of Pediatrics, Faculty of Medicine, Cairo University, Cairo, Egypt; 2 Department of Clinical Pathology, Faculty of Medicine, Cairo University, Cairo, Egypt

**Keywords:** Bone age, growth hormone therapy, Turner’s syndrome

## Abstract

**BACKGROUND AND OBJECTIVES:**

Recombinant human growth hormone (rhGH) is approved for use in children with Turner’s syndrome (TS) in most industrialized countries and is recommended in the recently issued guidelines. We determined the growth responses of girls who are treated with rhGH for TS, with an aim to identify the predictors of growth response.

**MATERIALS AND METHODS:**

Fifty-six prepubertal girls with TS, documented by peripheral blood karyotype, were enrolled. All the patients received biosynthetic growth hormone therapy with a standard dose of 30 IU/m^2^/week. The calculated dose per week was divided for 6 days and given subcutaneously at night.

**RESULTS:**

This study showed that rhGH therapy provides satisfactory auxological results. Bone age delay is to be considered as a predictive factor which may negatively influence the effect of rhGH therapy on final height. The growth velocity in the preceding year is the most important predictor of rhGH therapy response.

**CONCLUSION:**

These observations help us to guide rhGH prescription, to reduce the risks and costs.

## Introduction

Approximately, 1 in 2500 live female births is affected by Turner’s syndrome (TS), making it one of the more common genetic conditions encountered in pediatric practice. TS is caused by deletion of all (monosomy) or part (partial monosomy) of the second sex chromosome. Multiple body systems can be affected to varying degrees, presenting both diagnostic and management challenges for the pediatrician.[1] In recent years, knowledge and understanding of TS have advanced substantially. A Study Group recently published the guidelines for the care of girls and women with TS.[2] The diagnosis of TS requires the presence of characteristic physical features in phenotypic females, coupled with complete or partial absence of the second sex chromosome, with or without cell line mosaicism.[3]

Short stature is a hallmark of TS, with an estimated mean loss of 20 cm associated with X chromosome aneuploidy.[4] Studies of this condition have increased our understanding of the role of sex chromosomes in growth regulation and led to the discovery of the *SHOX* gene.[5] Short stature in TS is characterized by mild intrauterine growth retardation, slow growth during infancy, delayed onset of the childhood component of growth, and growth failure during childhood and adolescence without a pubertal growth spurt. This growth failure leads to a reduced final height.[6] Early attempts to increase stature by treatment with extracted growth hormone (GH) gave variable results. Analyses based on comparison with historical controls have shown variable estimates of gains in mean height, ranging from minimal in a study by Canadian investigators[7] to up to 17 cm for high doses of GH.[8]

It is important to remember, however, that up to 30% of girls with TS undergo spontaneous pubertal development, and 2–5% have spontaneous menses and may have the potential to achieve pregnancy without medical intervention.[9–12] Pubertal development may be delayed and, in most patients, is followed by progressive ovarian failure.[13]

Gonadal dysgenesis is a cardinal feature of TS; 90% of patients require hormone-replacement therapy to initiate puberty and complete growth. *In utero*, the ovaries have a decreased number of primordial follicles; these appear to undergo premature apoptosis[10] and are usually absent by adult life. The uterus may be small owing to lack of estrogen; structural uterine abnormalities are rare.

Initiation of GH therapy should be considered as soon as a patient with TS has dropped below the fifth percentile of the normal female growth curve. Therapy may be started as early as 2 years of age, although there is presently only limited experience of treating children of this age. GH therapy should be directed by a pediatric endocrinologist. Early GH treatment can correct the growth failure and normalize the height in infants and toddlers with TS.[14]

## Aim

In the present study, we determined the growth responses of girls treated with GH for TS, aiming to identify the predictors of growth response.

## Materials and Methods

A prospective cohort study on short statured, prepubertal TS cases was done. Their demographic, anthropometric and hormonal factors which may be related to their therapeutic response to GH were analyzed. Also, the effect of therapy on GH mediators, such as IGF-I and IGF-I BP3, in the same group of patients was evaluated.

### 

#### Patients

Fifty-six prepubertal girls with TS, documented by peripheral blood karyotype, were enrolled. Inclusion criteria were euthyroid TS cases having short stature, more than 2SD below the mean, or growth velocity (monitored over 6–12 months) below the 10^th^ centile for age and sex. The exclusion criteria were phenotypic females with identifiable Y chromosome material, cases with chronic diseases or any relevant cardiac or renal abnormalities, and those who have had prior treatment with GH. The study was done at Cairo University Children’s Hospital and National Research Centre. Cases were followed up for a minimum period of 1 year and for a maximum of 4 years.

#### Methods

Informed consent was taken from the parents of children; then all cases were subjected to the following. Full history taking and clinical examinations were done. Full anthropometric assessment was also done, including target and mid-parental heights. Target height was calculated by the method of Tanner *et al*., taking the average of mother’s and father’s height after subtraction 13 cm from the average, while mid-parental height was calculated as before ±6.5 cm.[15]

Height was measured twice and neared to the next millimeter using Harpenden Stadiometer height velocity in cm/year is the variable that describes the patient’s 1 year velocity and plotting it in the mid-year interval. Sitting height was also measured using Harpenden sitting height apparatus. Lower segment was calculated by subtraction of sitting height from height, and then from these two measurements, upper to lower segment ratio was derived (US/LS).

Weight of the patients was measured using electronic balances and recorded in decimal of kilogram. Puberty was assessed by rating the breast development in girls, pubic and axillary hair development, according to Tanner’s classification.[16] All anthropometric procedures were performed at baseline before treatment and at follow-up by the same observer at the same time of the day (9 a.m.–1 p.m.) in the growth clinic of the Diabetes Endocrine Metabolism Pediatric Unit (DEMPU) at Cairo University Children’s Hospital.

All auxological data were expressed in standard deviation scores (SDS) for TS patients. Standards for height and height velocity, specific for TS were used.[17] All the auxological data including estimated mature height (EMH) were analyzed by a software program (growthvision.2) provided by Novo–Nordisk, Denmark.

Skeletal maturity was determined by the same observer from an X-ray of the left wrist and hand (Tanner Whitehouse no.2 method). Bone age delay, delta bone age and EMH were derived.[18]

#### Laboratory investigations included

1. *Thyroid profile* (FT3, FT4, TSH) was done to exclude primary or secondary hypothyroidism as a cause of short stature. Thyroid stimulating hormone (TSH) was estimated by immunoradiometric assay (IRMA), while FT3 and FT4 were estimated by radioimmunoassay kits from Diagnosis Product Corporation, (Los angeles, CA, USA.)
2. *Routine general laboratory tests*, if needed, which include complete blood picture, renal and liver function tests.
3. Insulin like growth factor-1 (IGF-1) and IGF binding protein-3 (IGFBP-3) were determined at diagnosis, by solid phase IRMA, using kits from Diagnostic System Laboratories Inc. (Webster, TX, USA). DSL-5600 IGF-1 (IRMA) was included in a sample extraction step in which IGF-1 was separated from its binding protein in serum. This step is considered to be essential for accurate determination of IGF-1.[19]

#### Treatment protocol

All the patients received biosynthetic GH therapy. Patients with TS were treated with a standard dose of 30 IU/m^2^/week. The calculated dose per week was divided for 6–7 days and given subcutaneously at night. Puberty was not induced by giving sex hormones during GH treatment, since the treatment was started relatively late in these patients. All the patients accepted postponing induction of puberty after explanation by the physician.

#### Follow-up

Patients were followed for a minimum period of 1 year and for a maximum of 4 years.

The study group was followed up every 3 months for anthropometric assessment to assure compliance to therapy, to observe side effects and to renew the GH prescription. Follow-up of thyroid profile, IGF-1 and IGFBP-3 was done every 6 months and that of skeletal maturity was done every year in the hospital.

Every year, the surface area of each patient was calculated, and the dose of GH was adjusted to keep the therapeutic dose at 30 IU/m^2^/week (equivalent to 0.3 mg/kg/week) for TS. Response to GH therapy was judged based on data obtained from auxological assessment, skeletal maturity, and EMH.

Compliance to therapy was continuously verified by more than one parameter, e.g. height velocity, asking the parents about mode of injection and dosing, counting the empty vials and sometimes by the analysis of serum IGF-1.

The decision to stop GH treatment was made when the final adult height criteria was fulfilled and these included full pubertal development (Tanner stage 5), complete fusion of the epiphysis and growth velocity < 1 cm/year in the last year. Near final adult height is defined when the following criteria were achieved: Tanner stage 4 or more and bone age at least 14 years for females.

The deviation of individual IGF-1 and IGFBP-3 values from the means for age and sex was calculated in standard deviation score (*Z* score) and subsequently used in statistical analysis. The laboratory of DEMPU, Cairo University children’s Hospital, provided the mean values for IGF-1 and IGFBP-3.

#### Statistical analysis

The SPSS software computer program was used for data analysis, and Harvard graphic was used for figures. Quantitative data were presented as mean ± SD, range and frequencies, and qualitative data were presented as percentage. For comparison of the two groups, the Student’s *t*-test for dependent and independent variables, was used. For comparison of more than two groups, one-way analysis of variance (ANOVA) was used, followed by *post hoc* test, when significant. *P* value was considered significant if it was less than or equal to 0.05. For better evaluation of growth response during the 4 year study period, we included patients who had complete anthropometric data during this period, and delta changes for all the parameters were compared.

Linear Pearson correlation was done, followed by multiple linear regression, where the *r* value <0.2 was considered weak correlation, 0.2–0.5 was considered mild correlation, 0.5–0.8 was considered moderate correlation, and if *r* > 0.8, it was considered strong correlation.

## Results

The fifty-six patients who participated in this study had a mean age at onset of 8.8 ± 3.0 years, chronological age (CA) at onset of therapy 12.7 ± 2.4 years, BA at onset of therapy 10.5 ± 2.2 years and BA delay of 2.1 ± 1.4 years. The duration of delay of treatment (years) was 3.9 ± 2.4 years in TS.

Basal auxological data were as follows. Height SDS was –4.3 ± 0.9 in TS and weight SDS was –2.3 ± 1.3. Weight for height SDS was 1.9 ± 2. US/LS SDS was 1.8 ± 1.8.Triceps SFT SDS was –0.1 ± 1.5. Subscapular SFT SDS was 0.7 ± 1.8.

Descriptive statistics are presented in Table 1 for female patients with TS.

**Table 1:** Anthropometric, skeletal maturity and laboratory data of patients with Turner’s syndrome

Variables	Basal	First year	Second year	Third year	Fourth year
	
	Mean ± SD (n = 56)	Mean ± SD (n = 56)	Mean ± SD (n = 20)	Mean ± SD (n = 6)	Mean ± SD (n = 3)
Height (SDS) Tanner	–4.3 ± 0.9	–3.8 ± 1.5	–3.4 ± 0.9	–3.3 ± 0.7	3.0 ± 0.9
Height (SDS) Ranke	–1.0 ± 0.8	–0.5 ± 0.9	0.2 ± 1.2	0.2 ± 0.7	
Growth velocity (cm)		6.1 ± 1.9	4.9 ± 1.7	4.9 ± 1.4	5.3 ± 0.7
Growth velocity (SDS) Tanner		2.0 ± 1.9	1.3 ± 2.2	0.8 ± 2.1	0.2 ± 2.3
Growth velocity (SDS) Ranke		2.1 ± 1.8	2.2 ± 1.6	1.9 ± 0.9	
Target height – height (cm)	32.8 ± 9.8	27.1 ± 9.2	19.9 ± 8.2	17.0 ± 4.1	12.9 ± 2.5
Target height – height (SDS) Tanner	3.5 ± 1.2	3.2 ± 1.1	2.8 ± 1.1	2.6 ± 0.6	2.3 ± 0.6
Target height – height (SDS) Ranke	0.4 ± 1.1	–0.1 ± 1.1	–0.7 ± 1.4	–1.1 ± 0.6	
Height gain (SDS) Tanner		0.5 ± 1.3*	0.3 ± 0.4**	0.2 ± 0.4***	0.1 ± 0.5****
Height gain (SDS) Ranke		0.5 ± 0.4	0.4 ± 0.5	0.3 ± 0.2	
EMH (cm)	144.6 ± 8.3	146.3 ± 8.2	144.7 ± 7.1	150.7 ± 4.5	152.1 ± 4.9
IGF-1 (SDS)	–0.4 ± 0.9	–0.2 ± 1.1	–0.5 ± 0.7	–0.4 ± 0.6	–0.8 ± 0.1
IGFBP-3 (SDS)	–0.7 ± 1.1	0.3 ± 1.3	0.6 ± 0.7	1.1 ± 1.6	0.01 ± 0.6
Delta BA/CA		0.8 ± 0.2	0.9 ± 0.1	1.1 ± 0.1	0.9 ± 0.3

* Height first year – height basal;

** height second year– height first year;

*** height third year – height second year;

**** height fourth year – height third year;

EMH – estimated mature height

**Figure 1:**
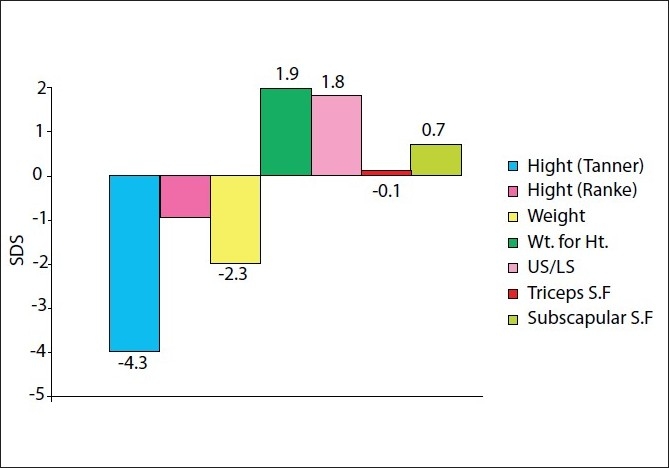
Basal auxological data in cases.

Follow-up was achieved for 56 patients in the first year, 20 patients in the second year, 6 patients in the third year and 3 patients were followed up in the fourth year. Turner patients showed some degree of disproportion where their US/LS ratio SDS was 1.8 ± 1.8 [Figure 1].

**Figure 2:**
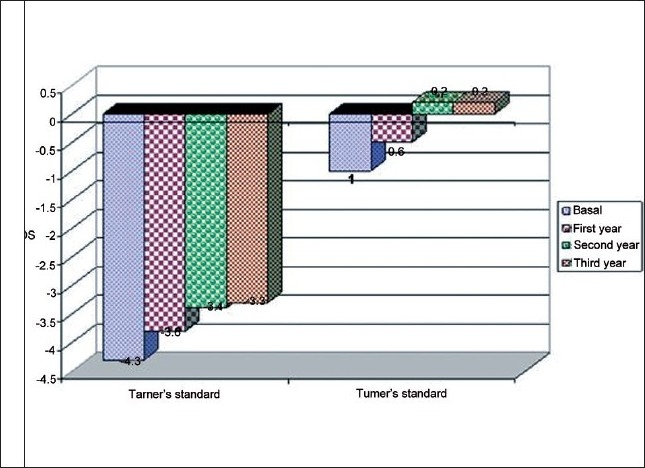
Height (SDS) follow-up in TS cases.

**Figure 3:**
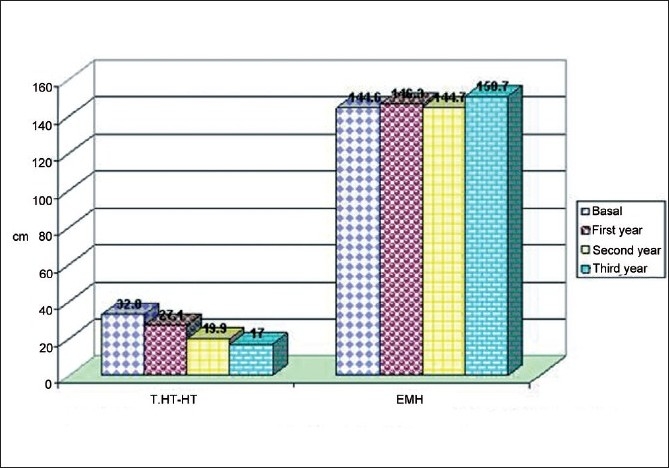
Target height – height (cm) and EMH in TS cases.

Growth response was as follows. Height SDS improved from –4.3 to –3.3 using Tanner standard and from –1 to 0.2 using Turner standard [Figure 2]. The patient’s height became much closer to the target height as it changed from 32.8 to 17 cm (3.5 to 2.6). EMH in TS improved from 144.6 to 150.7 cm [Figure 3].

**Figure 4:**
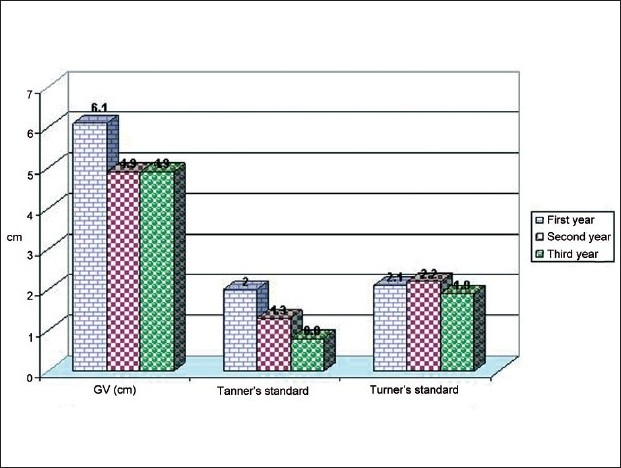
Follow-up growth velocity (cm and SDS) in TS cases.

The growth velocity in TS decreased from 6.1 cm (2 and 2.1 SDS using Tanner and Turner standards, respectively) to 4.9 cm (0.8 and 1.9 SDS using Tanner and Turner standards, respectively) [Figure 4].

Delta changes of 20 patients with TS, followed up for 2 years, showed a significant difference for target height – patient’s height in cm (*P* value = 0.002). [Table 2] The mean age of start of spontaneous puberty was 11.9 ± 1.3 years.

**Table 2:** Delta change of auxological and laboratory data of patients with Turner’s syndrome

Variables	First year	Second year	*P*-value
		
	Mean ± SD N=20	Mean ± SD N=20	
BA/CA	0.9 ± 0.2	0.9 ± 0.1	0.8
Height SDS	0.5 ± 0.3	0.4 ± 0.4	0.2
Target height - height (cm)	-6.5 ± 2.1	-5.1 ± 1.7	0.002**
Target height (SDS) - height (SDS)	-0.4 ± 0.3	-0.4 ± 0.4	0.4
EMH (SDS)	1.9 ± 2.5	2.3 ± 2.5	0.6
IGF-1 (SDS)	-0.4 ± 0.5	-0.8 ± 1.4	0.04*
IGFBP-3 (SDS)	1.8 ± 1.4	-1.5 ± 1.8	0.002**

EMH: estimated mature height; *P* value is significant if <0.05

### 

#### Prediction models

The regression equation for the first 3 years of treatment of TS is summarized in Table 3. In TS, the *r*^2^ was 0.61, 1 and 1 with SD error (cm) of 1.9 for the first, second and third years, respectively.

**Table 3:** Regression equations for prediction of growth response in girls with Turner’s syndrome

Variables	First year			Second year			Third year		
	Parameter estimar	Ranke	Percentage variability	Parameter estimar	Ranke	Percentage variability	Parameter estimar	Ranke	Percentage variability
Interapt (constant)		12.08			-2.38			-1.15	
Target height (SDS)				0.16	2	9			
BA delay (year)	-0.27	1	4						
IGF-I (SDS)				1.53	3	4			
1^st^ year GV (em/year)				0.88	1	49			
2^nd^ year GV (cm/year)							0.73	1	4
r^2^		0.61			1			1	
Error SD (cm)		1.9							

#### Insulin like growth factor-1 and its binding protein-3

IGF-1 SDS in TS [Table 1] raised from –0.4 ± 0.9 at the onset of GH therapy to –0.2 ± 1.1 after 1 year, and IGFBP-3 SDS increased from –0.7 ± 1.1 to 0.3 ± 1.3.

Only 20 patients with TS were followed up for 2 years [Table 2]; in these patients, the delta changes of IGF-1 SDS and IGFBP-3 showed significant difference with *P* values of 0.04 and 0.002, respectively.

## Discussion

Many patients with TS may not be the shortest child in kindergarten but will have a significant decrease in linear growth rate by third or fourth grade. Some present only when the normal pubertal growth spurt fails to occur.[20]

Our patients had a mean age at onset of diagnosis of 8.8 ± 3.0 years and mean CA at the onset of therapy of 12.7 ± 2.4 years, with a mean delay of treatment 3.9 ± 2.4 years. It is easy to misinterpret the absence of puberty and small size of these patients as due to constitutional delay.[20]

Turner patients showed some degree of disproportion where their US/LS ratio SDS was 1.8 ± 1.8 [Figure 1], which may give an evidence for an abnormality of the skeletal and/or growth plate as a cause of short stature. Being a type of skeletal dysplasia was previously suggested by Bridges and Brook;[21] however, X-rays lacked convincing evidences for skeletal dysplasia.[22]

GH is approved for use in children with TS in most industrialized countries and is recommended in the recently issued guidelines.[23] Numerous studies have reported that recombinant human growth hormone (rhGH), with or without anabolic steroids, can accelerate growth and lead to a height greater than that predicted in girls with TS.[24–28] In one cohort, the mean height of girls who completed a mean of 7.6 years of rhGH therapy (*n* = 17) was 150.4 cm, a gain of 8.4 cm over the expected average height.

The Canadian randomized control trial was published recently. They randomly assigned 154 children to treatment with GH or no treatment and both the groups were observed until the adult height was reached. They were able to follow-up 61 of 76 treated patients and 43 of 78 controls to adult height and observed a 7.2 cm difference between the two groups, with a 95% confidence interval of 6.0–8.4 cm. The mean duration of treatment was 5.7 years, and puberty was induced pharmacologically at a chronological age of 13 years.[28]

Growth response of our cases to GH showed that height SDS improved from –4.3 to –3.3 using Tanner standard and from –1 to 0.2 using Turner standard [Figure 2].

The patient’s height became much closer to the target height as itchanged from 32.8 to 17 cm (3.5 to 2.6). Estimated mature height in TS improved from 144.6 to 150.7 cm [Figure 3].

The growth velocity in TS decreased from 6.1 cm (2 and 2.1 SDS using Tanner and Turner standards, respectively) to 4.9 cm (0.8 and 1.9 SDS using Tanner and Turner standards, respectively) [Figure 4].

Delta changes of 20 patients with TS, followed up for 2 years, showed a significant difference for target height – patient’s height in centimeters (*P* = 0.002) [Table 2].

In the first year, the most important predictive factor was BA delay (negative correlation), which explained 61% of the variability in response with an error SD of 1.9 cm. For the second year, growth velocity of the first year was the strongest predictive factor (positive correlation), followed by target height SDS (negative correlation) and IGF-1 SDS (positive correlation). For the third year, growth velocity of the preceding years was the most important predictive factor (positive correlation). Predictive factors in the second and third years of GH therapy explained all of the variability in the response. So, it is apparent that during the period of catch up growth in girls with TS, BA delay was the most important predictor; the more the delay, the better is the growth response, giving a potential chance for better growth. During the period of stable growth, the growth velocity in the preceding year is the most important predictor; the higher the GV in the preceding year, the better is the response.

The growth responses of 686 girls with TS were determined during the first4 years of GH therapy. For the first year, GH dose was the most important predictor (positive correlation). Age, weight SDS, additional treatment with oxandrolone, the difference between the height SDS and mid-parental height SDS and frequency of injections were other predictors. These variables explained 46% of the variability of the response with an error SD of 1.26 cm. Height velocity in the preceding year was the most important predictor in the second to fourth year, with GH dose, age, weight SDS and addition of oxandrolone to treatment as the other predictors.[29] It was further reported that although the accuracy of prediction in all4 years was high, indicated by the low error SDS, the predictive power was relatively low.[30–31] In contrast to this result, we reported a high predictive power in the second and third years where the predictive factors explained all the variability in response to GH treatment in TS. In disagreement with this study, GH dose (dose/week), frequency of GH injections and birth weight were not included as variables in the present work. Also, none of our patients received oxanodrolone therapy. Otherwise, predictive variables were similar. In the present study, we had only one girl with TS who reached FAH; she had a height of 143.5 cm (3.1 SDS). A final adult height that varies between 147.7 ± 5.6 and 152.3 ± 5.3 has been reported in girls with TS. Monitoring of IGF-1 and IGF-BP3 levels in girls with TS during the initial 3 years of GH therapy showed a significant rise of IGF-1 values in the first 2 years, whereas the IGF-BP3 values showed a significant rise in the first year followed by a significant drop in the second year. The significant rise of growth factors IGF-1 and its binding protein-3 during early period of GH treatment, which is followed by their decline, may be related to the initial catch up growth which is followed by a steady pattern on the continuation of GH treatment.

In conclusion, this study seems to show that GH therapy provides a satisfactory auxological result. Bone age delay is to be considered as a predictive factor that may negatively influence the effect of GH therapy on final height. The growth velocity in the preceding year is the most important predictor of GH therapy response.

These observations help to guide rhGH prescription to reduce the risks and costs.
